# Neuroprotective effects of ATPase inhibitory factor 1 preventing mitochondrial dysfunction in Parkinson's disease

**DOI:** 10.1038/s41598-022-07851-8

**Published:** 2022-03-09

**Authors:** InHyeok Chung, Han-A Park, Jun Kang, Heyyoung Kim, Su Min Hah, Juhee Lee, Hyeon Soo Kim, Won-Seok Choi, Ji Hyung Chung, Min-Jeong Shin

**Affiliations:** 1 Interdisciplinary Program in Precision Public Health, Korea University, Seoul, Republic of Korea; 2 Department of Integrated Biomedical and Life Science, Korea University, Seoul, Republic of Korea; 3 Biotechnology Research Center, MediandGene Inc., Seoul, Republic of Korea; 4 Department of Human Nutrition and Hospitality Management, College of Human Environmental Sciences, The University of Alabama, Tuscaloosa, USA; 5 Department of Biotechnology, CHA University, Pocheon, Republic of Korea; 6 School of Biological Sciences and Technology, College of Natural Sciences, Chonnam National University, Gwangju, Republic of Korea; 7 Department of Anatomy, Korea University College of Medicine, Seoul, Republic of Korea; 8 School of Biosystems and Biomedical Sciences, College of Health Science, Korea University, Seoul, Republic of Korea

**Keywords:** Parkinson's disease, Cell death in the nervous system

## Abstract

Mitochondrial dysfunction is a key element in the progression of Parkinson’s disease (PD). The inefficient operation of the electron transport chain (ETC) impairs energy production and enhances the generation of oxidative stress contributing to the loss of dopaminergic cells in the brain. ATPase inhibitory factor 1 (IF1) is a regulator of mitochondrial energy metabolism. IF1 binds directly to the F_1_Fo ATP synthase and prevents ATP wasting during compromised energy metabolism. In this study, we found treatment with IF1 protects mitochondria against PD-like insult in vitro*.* SH-SY5Y cells treated with IF1 were resistant to loss of ATP and mitochondrial inner membrane potential during challenge with rotenone, an inhibitor of complex I in the ETC. We further demonstrated that treatment with IF1 reversed rotenone-induced superoxide production in mitochondria and peroxide accumulation in whole cells. Ultimately, IF1 decreased protein levels of pro-apoptotic Bax, cleaved caspase-3, and cleaved PARP, rescuing SH-SY5Y cells from rotenone-mediated apoptotic death. Administration of IF1 significantly improved the results of pole and hanging tests performed by PD mice expressing human α-synuclein. This indicates that IF1 mitigates PD-associated motor deficit. Together, these findings suggest that IF1 exhibits a neuroprotective effect preventing mitochondrial dysfunction in PD pathology.

## Introduction

Parkinson’s disease (PD) is a neurodegenerative disease characterized by motor symptoms including tremor, bradykinesia, rigidity, postural instability^1^, and non-motor symptoms such as depression, anxiety, and sleep disturbances^2,3^. A loss of dopaminergic neurons (DA) in the substantia nigra (SN), a part of the midbrain, is highly associated with PD pathology^4,5^. Although multiple variables, including genetic and environmental factors contribute to the development of PD, mitochondrial dysfunction is a key intracellular event in the degeneration of dopaminergic neurons^6,7^.

Mitochondria play central roles in regulating neuronal energy metabolism via the electron transport chain (ETC). SN neurons from PD patients have displayed deficiencies in ETC complex I and complex II^8^ which generate the reactive oxygen species (ROS) and trigger gradual damage to dopaminergic neurons^9^. Targeting the ETC with neurotoxins such as 1-methyl-4-phenyl-1,2,3,6-tetrahydropyridine and rotenone mimic PD pathology^10^. Inefficient ETC operation simultaneously increases ROS production and eventually makes cells susceptible to the collapse of mitochondrial membrane potential with impeded proteostasis by which they initiate intrinsic cellular death signals^11^. Moreover, accumulated ROS in PD pathogenesis has also been connected to deletion in mtDNA^12^ and subsequent ETC disruption forming viscous circle. Considering the recent evidence suggesting the role of mitochondrial function-related genes, such as autosomal recessives of *Parkin*, *PINK1*, and *ATP13A2* in PD etiology^13^, maintaining mitochondrial function is closely associated with PD pathophysiology.

ATPase inhibitory factor 1 (IF1) is a major player in neuronal energy metabolism^14,15^. IF1 is a regulatory protein that occupies space between the α and β subunits of the F_1_Fo ATP synthase, and this interaction inhibits both synthase and hydrolase activities^16^. The dimeric IF1 binds to the F_1_Fo ATP synthase in the acidic microenvironment, whereas IF1 forms inactive tetramers when pH increases^17^. Although the exact mechanisms of IF1 in mitochondrial function are still disputed, its role in inhibiting the hydrolytic activity of the F_1_Fo ATP synthase helps cells prevent ATP wasting during de-energized conditions^18–20^. IF1 is reported to prevent remodeling of mitochondrial structure and mitochondria-mediated apoptotic pathways that promote survival of various cancer cells^21,22^. In recent decades, an increasing number of reports have suggested a protective role for IF1 in neurodegeneration^23,24^. IF1 is necessary for mitochondrial translocation of PTEN-induced putative kinase (PINK-1)^23,25^, an enzyme encoded by the *PARK6* gene. PINK-1 is an important player during mitophagy by its role recruiting ubiquitin E3 ligase, Parkin. Impaired PINK-1 expression or activities is associated mitochondrial dysfunction in Parkinson’s diseases^26^. In addition to its role in regulating the mitochondrial population, IF1 controls energy metabolism in the brain. Genetically modified mice expressing IF1 shifted energy metabolism from oxidative phosphorylation to glycolysis, and this metabolic reprogramming protected the brain against apoptosis^27^.

In this study, we found that IF1 rescues dopaminergic cells from rotenone-induced loss of cellular energy, generation of mitochondrial ROS, and activation of apoptosis in vitro. Furthermore, administration of IF1 improved motor function in a transgenic mouse model of PD. Our results suggest that IF1 exhibits neuroprotective roles in PD pathology by regulating mitochondrial function.

## Results

### Treatment with IF1 protects mitochondria in SH-SY5Y cells against rotenone challenge

**Figure 1:**
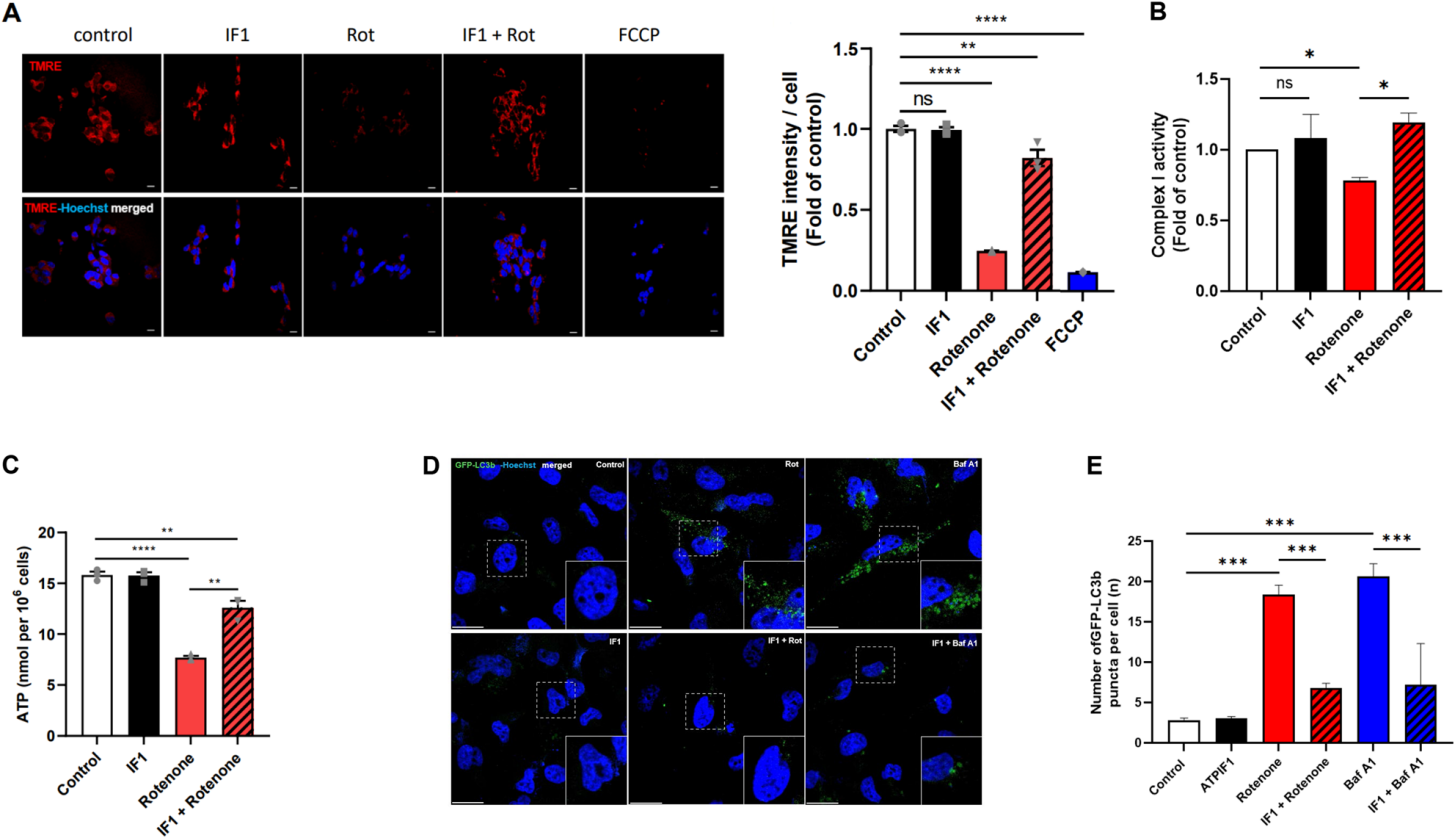
Treatment with IF1 protects mitochondria against a PD-like challenge in vitro. SH-SY5Y cells were treated with 1 µM rotenone, 100 nM IF1, or a combination of both for 24 h. (**A**) Representative confocal images of TMRE stained SH-SY5Y cells. Red: TMRE; Blue; Hoechst. Scale bar = 20 μm. A bar graph indicates the quantification of mean fluorescence intensity of TMRE normalized to the number of cells in each group. (**B**) SH-SY5Y cells were treated as indicated, and Complex I activity was detected and presented as a bar graph. (**C**) SH-SY5Y cells were treated as indicated, and intracellular ATP level was detected and presented as a bar graph. (**D**) GFP-LC3B expressing SH-SY5Y cells were treated with 1 µM rotenone, 100 nM IF1, 10 nM bafilomycin A1 (Baf A1) or a combination of them for 24 h. Green: GFP-LC3; Blue: Hoechst 33,342. Scale bar = 20 μm. (**E)** A bar graph indicates the number of GFP-LC3B puncta per cell in each group. Data are expressed as mean ± SEM, n = 3. *p < 0.05, **p < 0.01, ***p < 0.001, ****p < 0.0001. *ns* not significant. One-way ANOVA with a Tukey’s post-hoc analysis.

Due to its role in inhibiting mitochondrial apoptosis, we tested if treatment with IF1 protects mitochondria against a PD-like challenge in vitro. For the last of study, we selected SH-SY5Y Human neuroblastoma cell line to explore the role of IF1 in DA neurons. First, Mitochondria were stained with tetramethylrhodamine ethyl ester (TMRE), a cationic dye that visualizes mitochondrial inner membrane potential. Rotenone significantly decreased TMRE-positive fluorescence (Fig. 1A; Supple 1A). Since rotenone targets complex I blocking maintenance of the proton gradient, decreased levels of mitochondrial inner membrane potential were expected. Treatment with IF1, however, prevented rotenone-mediated loss of mitochondrial inner membrane potential (Fig. 1A). Maintaining mitochondrial inner membrane potential is pivotal to generate ATP by the ETC and F_1_Fo ATP synthase. Therefore, we tested whether IF1 can rescue the Rotenone-induced Complex I failure while quantified intracellular ATP levels to evaluate mitochondrial function (Fig. 1B,C). Interestingly, SH-SY5Y cells challenged with rotenone decreased both Complex I activity (Fig. 1B) and intracellular ATP levels (Fig. 1C) at 24 h, whereas treatment with IF1 prevented the loss of function and ATP level. Cellular energy deficit can occur during both mitochondrial dysfunction and loss of mitochondrial population. Studies demonstrated that insufficient and excessive autophagy are found in PD patients^28,29^, thus, we tested whether IF1 plays a role in regulating mitochondrial degradation. SH-SY5Y cells overexpressing GFP-LC3 demonstrated that treatment with rotenone and known autophagy inhibitor bafilomycin A 1 (Baf A1) enhanced autophagosome accumulation (Fig. 1D,E), whereas treatment with IF1 decreased GFP-LC3 puncta indicating a potential role preventing aberrant mitophagy.

### Treatment with IF1 decreases oxidative stress in SH-SY5Y cells against rotenone challenge

**Figure 2:**
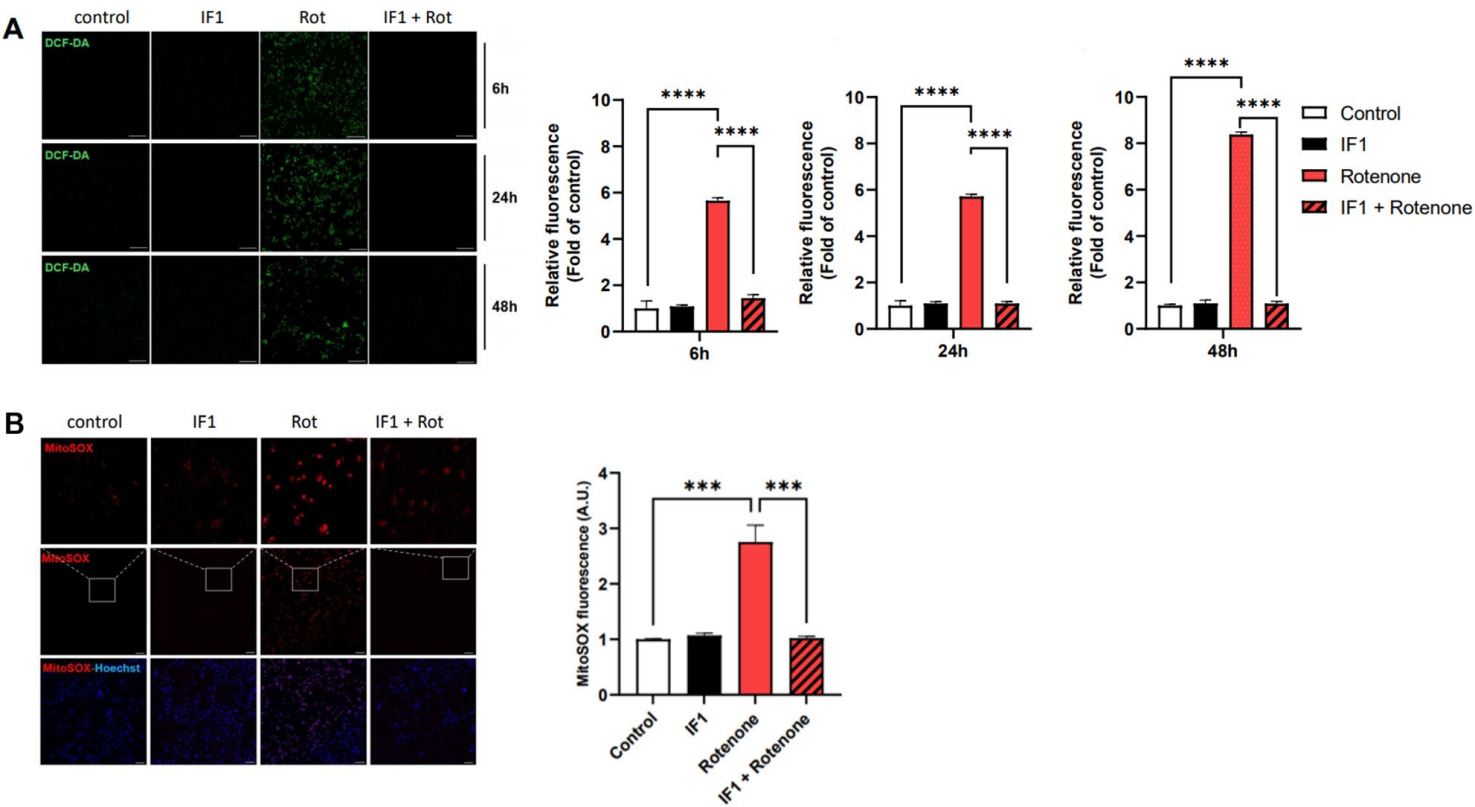
Treatment with IF1 prevents rotenone-induced ROS generation in SH-SY5Y cells. SH-SY5Y cells were treated with 1 µM rotenone, 100 nM IF1, or a combination of both. (**A**) Representative images of ROS level using DCF-DA in cultured SH-SY5Y cells (right panel) and quantification of fluorescence was analyzed at 6 h, 24 h, and 48 h (left panel). Scale bar = 200 μm. (**B**) Representative confocal images of mitoSOX staining in SH-SY5Y cells (left pane) and fluorescence were quantified (right panel). Scale bar = 200 μm. Data are expressed as mean ± SEM, n = 3. **p < 0.01, ***p < 0.001, ****p < 0.0001. One-way ANOVA with a Tukey’s post-hoc analysis.

Failure to maintain mitochondrial inner membrane potential and inefficient operation of the ETC results into the generation of ROS^30^. Thus, we quantified endogenous peroxide levels in SH-SY5Y cells using 2′,7′-dichlorofluorescein (DCF). SH-SY5Y cells treated with rotenone significantly increased DCF fluorescent intensity, and fluorescent signal remained high until 48 h indicating prolonged exposure to oxidative stress (Fig. 2A). However, the intracellular ROS level was largely decreased in the IF1 co-treated group. We further labeled SH-SY5Y cells with mitoSOX, a fluorescent probe to visualize mitochondrial superoxide. Consistently, cells treated with rotenone demonstrated significantly increased mitoSOX fluorescence, whereas IF1 co-treatment decreased rotenone-induced mitoSOX fluorescence indicating inhibition of mitochondrial superoxide generation (Fig. 2B).

### Treatment with IF1 protects dopaminergic cells against PD-like insults in vitro

**Figure 3:**
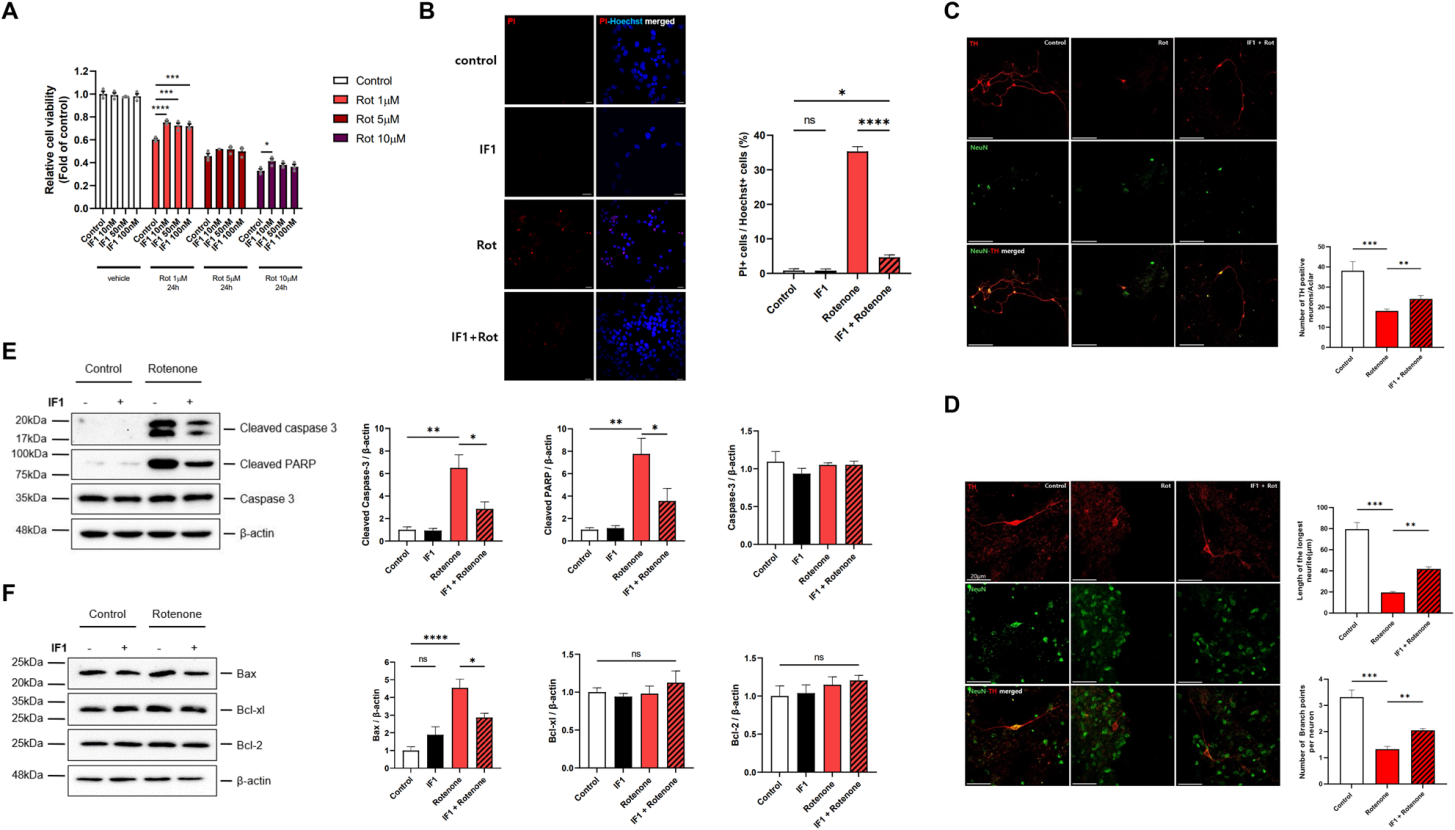
Treatment with IF1 protects dopaminergic cells from PD-like neurotoxicity and prevents rotenone-induced apoptosis in SH-SY5Y cells. **(A**) SH-SY5Y cells were treated with rotenone (1, 5, and 10 µM) with or without a specific concentration of IF1 (10, 50, and 100 nM). After 24 h incubation, cell viability was evaluated by MTT assay. (**B**) SH-SY5Y cells were treated with 1 µM rotenone, 100 nM IF1, or a combination of both for 24 h. Representative images of propidium iodide (PI) staining results. Red: PI; Blue: Hoechst, Scale bar = 20 µm (left panel). A bar graph indicates the number of PI positive cells normalized to Hoechst in each group (right panel). (**C**) Mouse primary dopaminergic neurons were treated with 1 µM rotenone with or without 10 nM IF1 for 16 h and simultaneously immunostained for tyrosine hydroxylase (TH) and Neun (Neuronal marker). The number of TH-positive cells was observed (upper panel), quantified, and presented as a bar graph (lower panel). Scale bar = 100 μm. (**D**) The average length of longest neurites and the number of branches per neurites of TH positive cells were observed (upper panel), quantified, and presented as a bar graph (lower panel). Scale bar corresponds to 50 μm. One-way ANOVA with a Tukey’s post-hoc analysis. (**E,F)** SH-SY5Y cells were treated with 1 µM rotenone, 100 nM IF1, or a combination of both for 24 h. (**E)** The abundance of protein levels of cleaved caspase-3, cleaved PARP, and full-length caspase-3 were analyzed using immunoblotting (left panel) and quantified (right panel). (**F**) Protein levels of Bcl-2 family (Bax, Bcl-xL, and Bcl-2) were analyzed using immunoblotting (left panel) and quantified (right panel). All protein levels were normalized to β-actin and presented as a bar graph. **(E,F)** Membranes were cut to enable blotting for multiple antibodies and the full-length blots are presented in Supplementary information file. Data are expressed as mean ± SEM, n = 3. *p < 0.05, **p < 0.01, ***p < 0.001, ****p < 0.0001. *ns* not significant. One-way ANOVA with a Tukey’s post-hoc analysis.

Human neuroblastoma SH-SY5Y cells express tyrosine hydroxylase (TH), a key enzyme that converts tyrosine to dihydroxyphenylalanine (DOPA), and therefore, important during the production of neurotransmitters such as dopamine, norepinephrine, and epinephrine. SH-SY5Y cells also express dopamine receptors and transporters^31^, thus making them optimal in investigating PD pathology in vitro. We first screened viability of SH-SY5Y cells challenged with various concentrations (0.1, 0.2, 0.5, 1, 5, 10 μM) of rotenone (Fig. 3A; Supple 1B and Supple 2A), which inhibits ETC by blocking complex I. Higher concentrations of rotenone lowered the reduction of MTT by NAD(P)H oxidoreductase. Since viable cells continuously consume NADH in the ETC, our data indicate that treatment with rotenone causes loss of cell viability or alteration of cellular energy metabolism. We found that SH-SY5Y cells treated with 1 μM rotenone were consistently protected by multiple concentrations of IF1 (10, 50, and 100 nM). For the rest of study, we applied 1 μM rotenone and 100 nM IF1 to investigate the neuroprotective roles of IF1.

We analyzed cell death levels by measuring propidium iodide (PI), as a marker for late apoptosis and necrosis. SH-SY5Y cells challenged with rotenone significantly increased PI-positive cells, whereas treatment with IF1 prevented rotenone-induced cell death (Fig. 3B). Furthermore, we verified the neuroprotective effect of IF1 applying an additional dopaminergic cell line, MN9D murine neuroblastoma cells. Treatment with rotenone significantly decreased the viability of MN9D cells, whereas co-treatment with IF1 showed concentration-dependent protection against rotenone challenge (Supple 2B). Additionally, we challenged MN9D cells with 400 μM paraquat to mimic an environmental insult associated with PD. Treatment with IF1 protected MN9D cells against paraquat-mediated cytotoxicity (Supple 2C–E).

We validated the neuroprotective effects of IF1 using primary dopaminergic neurons isolated from mouse midbrain. TH-positive dopaminergic neurons were visualized by immunocytochemistry. Treatment with rotenone significantly decreased TH-positive cells indicating loss of dopaminergic neurons, whereas neurons treated with IF1 were protected (Fig. 3C). Finally, we tested the role of IF1 in regulating neuronal morphology by measuring the length and arborization of neurites. Neurite complexity achieved by neurite outgrowth and branching is critical to maintaining neuronal function and survival. We found that rotenone caused neurite pruning, but dopaminergic neurons treated with IF1 maintained their neurite morphology (Fig. 3D).

### Treatment with IF1 prevents rotenone-induced apoptosis in SH-SY5Y cells

To test if IF1-mediated neuroprotection is associated with its role in regulating apoptotic death signaling, we quantified apoptotic proteins. Treatment with rotenone caused activation of caspase-3 and cleavage of a downstream target of caspase-3, poly (ADP-ribose) polymerase (PARP) without changing protein levels of pro-caspase 3 (Fig. 3E). However, IF1 inhibited rotenone-induced apoptotic signaling decreasing the abundance of cleaved caspase-3 and cleaved PARP protein in rotenone challenged SH-SY5Y cells (Fig. 3E). Similarly, treatment with IF1 prevented paraquat-induced caspase-3 activation in MN9D cells (Supple 3).

Oligomerization of pro-apoptotic Bcl-2 proteins such as Bax on mitochondrial membranes contributes to caspase activation, whereas anti-apoptotic proteins such as Bcl-2 and Bcl-xL bind and sequestrate pro-apoptotic Bcl-2 proteins. Therefore, we tested whether IF1 can regulate the abundance of pro- or anti-apoptotic Bcl-2 family proteins (Fig. 3F). Treatment with IF1 significantly decreased protein levels of Bax against rotenone challenge (Fig. 3F).

### IF1 ameliorates motor deficits in α-synuclein transgenic PD mice model

**Figure 4:**
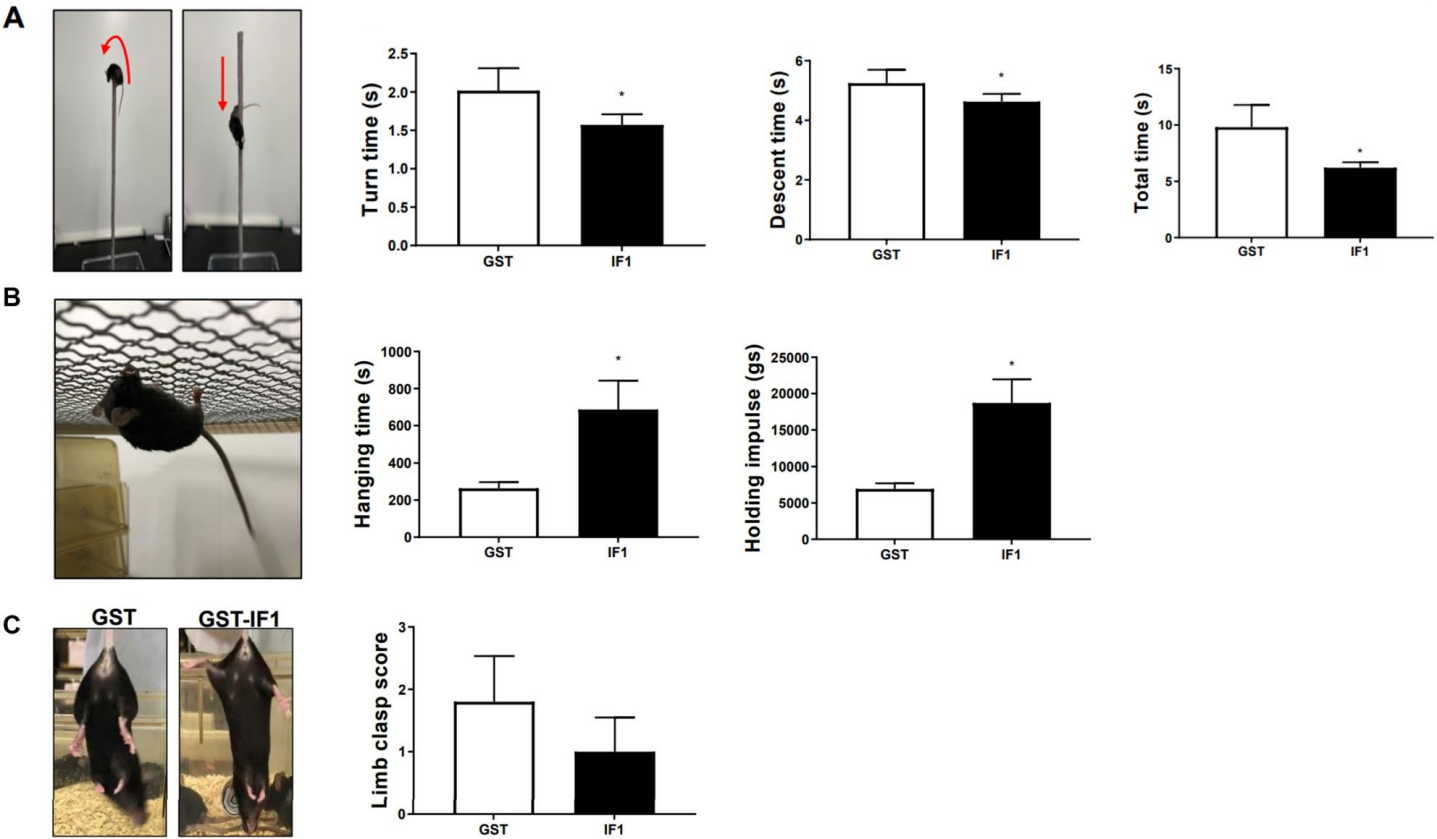
Administration of IF1 improves motor behavior of *α-syn* transgenic mice. Administration of IF1 in mice overexpressing α-synuclein was conducted to investigate the PD-like behavior abnormalities. (**A**) The pole test was conducted and each parameter, turn time, descent time, and total time, were compared within groups (GST, n = 5; IF1, n = 5). (**B**) Strength was assessed by hanging test. Hanging time and holding impulse from each group were evaluated. (**C**) Limb clasp performance was scored (left panel) and presented as a bar graph (right panel). Data are expressed as mean ± SEM, *p < 0.05. One-way ANOVA with a Tukey’s post-hoc analysis.

Given the protective effects of IF1 on dopaminergic neurons from neurotoxins, we explored the effect of IF1 in a transgenic PD mice model overexpressing α-synuclein, C57BL/6-Tg (NSE-hαSyn) Korl mice. Mice were intraperitoneally injected with IF1 (7.5 mg/kg) every other day for 9 weeks, and the control group was injected with the same concentration of the vehicle solution containing GST. After administration was complete, animals underwent behavioral tests to evaluate their motor functions. During the pole test, mice were placed on the top of the vertical pole, and their turn latency and descent latency were evaluated. Mice treated with IF1 significantly decreased turn, descent, and total test times (Fig. 4A) indicating improved motor coordination. Additionally, the hanging test was performed by assessing their hanging time and holding impulse on a wire grid. Consistently, mice treated with IF1 displayed significantly improved performance in the hanging test (Fig. 4B) implying that IF1 administration leads to recovering impaired motor function and movement symptoms in PD mice. Although it was not statistically significant, the mean limb clasp score in IF1 injected animals was lower than the control group (Fig. 4C).

## Discussion

In the present study, we found that treatment with IF1 protects mitochondria from PD-like insults and prevents depletion of energy, loss of mitochondrial potential, accumulation of ROS, and activation of apoptotic signaling in dopaminergic cells in vitro. Consistently, in vivo application of IF1 in transgenic PD mice resulted in improved motor behavior. It is well described that inefficient operation of the ETC contributes generation of mitochondrial ROS^32^. Numerous studies have demonstrated that treatment with rotenone elevates cellular ROS production by blocking complex I^33,34^, and our data consistently showed increased peroxide and superoxide levels in dopaminergic cells challenged with rotenone. We found that treatment with IF1 was effective in alleviating rotenone-induced ROS accumulation. IF1 showed no effect on ROS levels in SH-SY5Y cells without rotenone treatment. However, when cells were challenged with rotenone, IF1 significantly inhibited ROS generation at 24 h incubation, and cells maintained improved redox balance up to 48 h (Fig. 2A). Notably, treatment with IF1 completely reversed rotenone-induced production of mitochondrial superoxide suggesting mitochondria are the key target of IF1-mediated neuroprotection (Fig. 2B). Furthermore, IF1 prevented loss of mitochondrial inner membrane potential and conserved intracellular ATP, indicating improved mitochondrial function in SH-SY5Y cells during rotenone challenge. It was unexpected that IF1 alone did not alter total ATP level after 24 h incubation. We speculate that IF1 may protect the mitochondrial health from external toxins, yet not regulating the functionality of ETC beyond normal ranges. Additionally, primary cortical neurons isolated from transgenic mice expressing human IF1 did not show changes in mitochondrial membrane potential, but the levels of pyruvate kinase and lactate dehydrogenase were increased, suggesting enhanced glycolysis^27^. Although IF1 exhibited a minimal effect on healthy cells, treatment with IF1 may cause metabolic remodeling and support SH-SY5Y cells to maintain greater ATP. Therefore, it may be important to apply ETC and glycolysis inhibitors in future studies to distinguish the role of IF1 on mitochondrial vs. non-mitochondrial metabolism.

IF1-mediated mitochondrial protection ultimately rescues dopaminergic cells from rotenone-, or paraquat-indued cell death. Mitochondria are key players regulating apoptotic pathways. Both pro-apoptotic Bcl-2 proteins such as Bax and anti-apoptotic Bcl-2 proteins such as Bcl-xL and Bcl-2 are found in the mitochondrial membrane. During apoptotic signaling, pro-apoptotic Bax makes the mitochondrial membrane permeable, enhancing releases of cytochrome c to the cytoplasm. Cytochrome c forms an apoptosome activating executioner caspases including caspase-3. Rotenone was previously shown to activate apoptotic signaling in dopaminergic cells^35,36^. Consistently, we also found enhanced protein levels of Bax and the active form of caspase-3 in rotenone-treated SH-SY5Y cells. Treatment with IF1, however, significantly reversed activation of the rotenone-induced apoptotic pathway by inhibiting the formation of Bax. Since oligomerization of Bax contributes to loss of mitochondrial membrane potential, decreased levels of Bax may further protect the mitochondria. In the current study, we did not find IF1 had significant effects on anti-apoptotic Bcl-xL or Bcl-2 protein levels. Interestingly, studies have demonstrated that the loss of pro-survival effects among anti-apoptotic Bcl-2 proteins during post-translational modification. In particular, Bcl-xL undergoes N-terminal cleavage upon activation of caspase 3, and the cleaved form of Bcl-xL exhibits a pro-death effect^37–39^. An approach to inhibit the generation of ROS prevents the generation of the cleaved form of Bcl-xL^40^. Similarly, Bcl-2 is also subjected to caspase-3 and increases cytochrome c release indicating enhanced apoptosis^41^. Since we found activation of caspase 3 upon treatment with rotenone, SH-SY5Y cells challenged with rotenone may contain both anti-apoptotic and pro-apoptotic Bcl-xL or Bcl-2.

Various behavioral tests have been applied to characterize motor and non-motor phenotypes of PD models^42^. Mice with rotenone-induced PD exhibited abnormal locomotor behavior^43^. Transgenic mice expressing α-synuclein have shown impaired motor function as evaluated by the pole test, beam traversing test, and rotarod test^44,45^. In this study, we showed that administration of IF1 improved motor behavior of α-synuclein transgenic mice. Kinetics of IF1 delivery to the brain is still unknown, however, dysfunction of the blood–brain barrier (BBB) occurs during neurodegeneration, and PD pathology promotes BBB leakage. Thus IF1, a small protein (84-amino acid) may be delivered to the brain in PD mice. Additionally, we have previously reported that intraperitoneally delivered IF1 significantly decreased blood glucose levels in diabetic mice within 1 h via translocation of glucose transporter 4^46^. Since, glucose transporter 4 is found in neurons^47,48^, IF1 may also support neuronal energy metabolism by improving cellular update of glucose.

In conclusion, our study highlights the use of IF1 to counteract the neurotoxicity in dopaminergic neurons and uncover the mitochondria protective function in vitro. We further show an in vivo activity of IF1 that successfully confers positive effects on muscle strength and motor function in a transgenic PD mouse model.

## Materials and methods

### Reagents

The following chemicals and constructs were also used in this study: rotenone, tetramethylrhodamine ethyl ester (TMRE), Tris-buffered ATP, 4′,6-diamidine-2′-phenylindole dihydrochloride (DAPI), 2',7'-dichlorodihydrofluorescein diacetate (H2DCFDA), bafilomycin A1 (Baf A1) and carbonyl cyanide 4-(trifluoromethoxy)phenylhydrazone (FCCP) were purchased from Sigma-Aldrich (St. Louis, MO, USA). Paraquat, propidium iodide, and Hoechst 33342 were purchased from Thermo Fisher (Rockford, IL, USA).

### Cell culture and transfection

Human neuroblastoma SH-SY5Y cells and mouse dopaminergic MN9D cells were cultured in Dulbecco’s modified Eagle’s medium (DMEM; Invitrogen) supplemented with 10% fetal bovine serum (FBS) and 1% antibiotics at 37 °C in an atmosphere with 5% CO_2_. Cells were subcultured when 75% confluence was reached. *Drug treatment:* Cells were briefly washed with PBS and treated with IF1, rotenone, paraquat, or bafilomycin A1 (Baf A1) as described in relevant figure legend. A stock solution of rotenone and paraquat was prepared in DMSO. A control group was treated with the same volume of DMSO. *Transfection:* Transfection was performed using Lipofectamine 2000 (Thermo Fisher, Rockford, IL). SH-SY5Y cells were grown on the poly-d-Lysine coated 24-well plates and washed twice with DMEM on the 3rd day after plating. Lipofectamine (0.7 μl) in DMEM (50 μl) was mixed with GFP-LC3B plasmid DNA (300 ng) in DMEM (50 μl) for 30 min at RT. This mixture (100 μl) was dispensed into each well and incubated for 4 h. Prewarmed fresh medium was added to the cells and incubated for another 24 h.

### IF1-GST and GST protein purification

Recombinant GST-tagged IF1 and GST proteins were designed and purified as previously described^49^. Briefly, a pGEX vector containing the gene encoding human IF1 but without the signal peptide sequence was transformed into Escherichia coli strain BL21. Stable IF1-expressing cells were induced with 1 mM of isopropyl β-d-1-thiogalactopyranoside (IPTG) in lysogeny broth medium and lysed by probe sonication in phosphate-buffered saline (PBS). After centrifugation at 10,000×*g*, the protein-containing supernatant was collected and loaded onto a column containing glutathione Sepharose-4B resin (ELPIS-Biotech, Daejeon, Korea). The recombinant protein was eluted and dialyzed against PBS and verified using 10% sodium dodecyl sulfate (SDS)-polyacrylamide gel electrophoresis (PAGE).

### Mitochondrial membrane potential (ΔΨ) measurements

Changes in mitochondrial membrane potential were measured with TMRE. SH-SY5Y cells were plated in an 8-well chamber slide at a density of 30,000 per well and allowed to grow for 48 h. Then, the cells were washed twice with PBS and treated with 100 nM GST-IF1 and/or 1 μM rotenone for 24 h while 20 μM FCCP was used as a control treatment. The cells were incubated with TMRE 200 nM and 2.5 µg/ml Hoechst stain for 30 min before imaged using a confocal microscope (Model IX81 inverted; Olympus, Tokyo, Japan). TMRE intensity per cell was evaluated by using ImageJ software.

### Complex I activity assay

Evaluation of Complex I activity was performed using an Complex I Enzyme Activity Microplate Assay Kit (Colorimetric) (Abcam, Cambridge, UK) according to the manufacturer’s instructions. Briefly, 10,000,000 SH-SY5Y cells were lysed in detergent solution on ice, and lysates were subsequently centrifuged. The supernatant was transferred to new vial and normalized to their protein concentration. After diluting samples (5 mg/mL), samples were moved to a 96-well microplate and incubated for 3 h at room temperature. After washing 3 times with washing buffer, assay solution was added to each well and the absorbance was measured at 450 nm for 30 min with 1 min interval using a VICTOR X3 Multilabel Plate Reader (PerkinElmer, Waltham, MA, USA).

### Intracellular ATP assay

Evaluation of intracellular ATP level was performed using an ATP Colorimetric/Fluorometric Assay Kit (Biovision, Milpitas, CA, USA) according to the manufacturer’s instructions. Briefly, 100,000 SH-SY5Y cells were lysed with probe sonication in 100 μl of ATP assay buffer on ice, and lysates were subsequently moved to a 96-well microplate. Samples were then mixed with Reaction Mix for 30 min at room temperature, and the absorbance was measured at 570 nm using a VICTOR X3 Multilabel Plate Reader (PerkinElmer, Waltham, MA, USA). For converting absorbance to ATP concentration, an ATP standard curve was constructed using multiple concentrations of ATP solutions.

### Intracellular reactive oxygen species detection

#### H2-DCFDA staining

SH-SY5Y cells were cultured in an 8-well chamber slide and treated as indicated. After washing them twice with Hank’s Balanced Salt Solution (HBSS), cells were incubated with 20 μM H2-DCFDA in HBSS for 1 h. The excess probe was thoroughly washed with HBSS, and the solution was replaced by 25 mM hydroxyethyl piperazine ethane sulfonic acid (HEPES)-buffered HBSS. The live cells were imaged using a confocal microscope (Carl Zeiss). *mitoSOX staining:* Mitochondrial superoxide analysis was performed using the mitoSOX indicator (Invitrogen, Carlsbad, CA, USA) according to manufacturer’s instruction. In brief, SH-SY5Y cells cultured in an 8-well chamber slide were treated as indicated and washed with HBSS for twice. Cells were then following incubated with 2 μM MitoSOX-Red for 10 min 37 °C in a 5% CO_2_ atmosphere, washed with HBSS twice and mount in 25 mM HEPES buffered HBSS for fluorescence analysis. The live cells were imaged using a confocal microscope (Carl Zeiss).

### Cell viability assay

Cell viability was measured by MTT assay. First, cells were seeded on 96-well plate containing complete medium and cultured for 24 h at 37 °C in an atmosphere with 5% CO_2._ Then, cells were washed twice with PBS and treated with drugs as indicated. After 24 h stimulation, medium was removed, and the cells were washed twice with PBS. Finally, MTT dissolved in serum-free DMEM was added to dissolve the formazan crystals and the absorbance was measured at 570 nm using a VICTOR X3 Multilabel Plate Reader (PerkinElmer, Waltham, MA, USA).

### Propidium iodide (PI) staining

Cells were washed with warm PBS, stained with 250 μg/ml PI for 10 min, and fixed with 4% paraformaldehyde for 15 min at RT. The cells were washed thrice with PBS and stained with 2.5 μg/ml Hoechst for 10 min at RT. The cells were mounted and imaged using a confocal microscope (Model IX81 inverted; Olympus). The PI positive and Hoechst positive cells per 1 mm^2^ were counted and the percentage of PI positive/Hoechst positive cells were calculated.

### Primary mesencephalic neuron preparation

Embryonic day 14 (E14) mouse embryos were prepared from a C57BL/6N (Jackson laboratory) pregnant dam as described previously^50^. The dissected and dissociated tissues were incubated on a 10-mm-diameter ACLAR-embedded file precoated with 50 μg/ml PDL and 4 μg/ml laminin (BD Bioscience, Bedford, MA) in 24-well plates. Culture medium was added the next day and days in vitro at DIV 3. Half of the medium was replaced with an N2-supplemented medium (50% DMEM and 50% Hams F-12) at DIV 5–7. At DIV7, cells were treated with rotenone (1μΜ) alone or in combination with IF1 (10 nM) in N2-supplemented medium (50% DMEM and 50% Hams F-12) for 24 h.

### Western blotting

Cells were washed with PBS and lysed by radioimmunoprecipitation (RIPA) buffer supplemented with protease (Roche Applied Science, Mannheim, Germany) and phosphatase inhibitors (Sigma-Aldrich). Lysates were centrifuged at 13,000 rpm for 30 min, and the supernatant was collected. The resultant was collected and prepared by addition of 5× sample buffer, heated at 95 °C for 5 min, subjected to 8–15% SDS-PAGE, and analyzed by immunoblotting. Membranes were cut to enable blotting for multiple antibodies. The Primary antibodies used were as follows: caspase-3 (#9665), cleaved caspase-3 (#9664), cleaved PARP (#5625), and Bcl-xL (2764S) from Cell Signaling Technology (Danvers, MA); Bcl-2 (sc-783), Bax (sc-6236), and β-actin (sc-47778) from Santa Cruz Biotechnology (Dallas, TX).

### Immunocytochemistry

Cells were fixed with 4% paraformaldehyde for 30 min at RT and blocked in PBS containing 5% normal goat serum, 5% BSA, and 0.1% Triton X-100 for 30 min at RT. Primary antibodies were were a mouse monoclonal anti- tyrosine hydroxylase (1:3000, Sigma-Aldrich, St. Louis, MO, USA), a rabbit monoclonal NeuN (1:3000, abcam, Cambridge, UK) and a rabbit monoclonal caspase-3 (1:3000, Cell Signaling, Denvers, MA, USA). Cells were incubated with the antibodies in blocking buffer and incubated overnight at 4 °C. After 24 h incubation, the cells were washed thrice with PBS and incubated with Alexa 568-conjugated goat anti-rabbit IgG or Alexa 488-conjugated goat anti-rabbit IgG (1:5000) secondary antibody for 2 h at RT. Cells were then examined under a Zeiss inverted confocal microscope (Model LSM 700; Carl Zeiss Inc.). For quantification, at least 500 cells in each group were collected and measured in three independent experiments. The intensity of fluorescent was measured using NIH ImageJ software.

### Animal and study design

All mice had access to food and water ad libitum. The housing room temperature was maintained at 18–24 °C in simulated daylight conditions (12 h light/dark schedule). After 1 week of acclimatization, the animals were randomly divided into two groups: GST (n = 5) and GST-IF1 (n = 5). Each group of mice was intraperitoneally injected with the indicated proteins in every other day at a dose of 7.5 mg/kg for 9 weeks. At the end of the study, the mice were sacrificed, and the brains were extracted for further research. All mouse procedures were maintained according to the guidelines of the Korea University (Gyerim) Experimental Animal Resource Center. All studies involving mice were undertaken with approval from the Institutional Animal Care and Use Committee of Korea University (KUIACUC-2019-0059).

### Pole test

The pole test was used to evaluate movement disorders in PD mouse models. In this task, mice were placed head upward on the top of a wooden vertical pole (diameter 10 mm, height 55 cm), and trained to descend to their cage. Once placed on the pole, they oriented themselves downward (turn latency) to descend from the pole back into the ground (descent latency). Mice were trained for two days prior to testing, and the test was conducted in five consecutive trials. The turn latency and descent latency were averaged over training blocks. Mice displaying jumping or sliding behavior was scored as an error and excluded.

### Limb clasp test

The limb clasp task was adapted from a previously described protocol^51^. During the test, mice are suspended by the base of the tail and videotaped for 15 s. The mice were then placed back into their cages. Limb clasping was analyzed by inspecting the video and each subject was scored based on whether hindlimbs were splayed outwards or inwards. Specific scoring parameters are as follows: 0 = no limb clasping, normal escape extension, 1 = one hind limb incompletely retracted inwards, 2 = both hind limbs exhibited incomplete retraction inwards towards the abdomen, 3 = both hindlimbs completely retracted inwards with curled toes, 4 = forelimbs and hindlimbs exhibited complete retraction and crossing with curled toes. Three separate trials were performed two consecutive days.

### Hanging test

Neuromuscular abnormalities including muscle weakness and limb tension against gravity, were assessed using the hanging test. The test was conducted following a well-established grid hanging test (Carlson 2011). During the test, mice were placed on the top of the wire grid, which was then inverted and held at a height of 50 cm, enough to prevent the mouse from jumping down but not to cause harm from falling. Each mouse was placed in the same location and the time until fall was recorded. Average over total hanging time (s) besides holding impulse (body weight × hanging time; g × s) was presented as a correction to gravitational force.

### Statistical analyses

Researchers remained blinded throughout behavioral assessments. Groups were un-blinded at the end of each experiment during statistical analysis. The results of the experiments involving cell and animal systems are presented as means ± standard errors, and differences among the experimental groups were analyzed using Student’s t-test. The statistical analyses were performed using GraphPad Prism 9 software (GraphPad Software, La Jolla, CA, USA). A p-value of < 0.05 was considered statistically significant.

This study is reported in accordance with ARRIVE guidelines (https://arriveguidelines.org).

## Supplementary Information

Supplementary Figures.
