# Author Correction: Neuroprotective effects of ATPase inhibitory factor 1 preventing mitochondrial dysfunction in Parkinson's disease

**DOI:** 10.1038/s41598-022-09076-1

**Published:** 2022-03-25

**Authors:** InHyeok Chung, Han-A. Park, Jun Kang, Heyyoung Kim, Su Min Hah, Juhee Lee, Hyeon Soo Kim, Won-Seok Choi, Ji Hyung Chung, Min-Jeong Shin

**Affiliations:** 1 Interdisciplinary Program in Precision Public Health, Korea University, Seoul, Republic of Korea; 2 Department of Integrated Biomedical and Life Science, Korea University, Seoul, Republic of Korea; 3 Department of Human Nutrition and Hospitality Management, College of Human Environmental Sciences, The University of Alabama, Tuscaloosa, USA; 4 Department of Biotechnology, CHA University, Pocheon, Republic of Korea; 5 School of Biological Sciences and Technology, College of Natural Sciences, Chonnam National University, Gwangju, Republic of Korea; 6 Department of Anatomy, Korea University College of Medicine, Seoul, Republic of Korea; 7 School of Biosystems and Biomedical Sciences, College of Health Science, Korea University, Seoul, Republic of Korea

Correction to: *Scientific Reports* 10.1038/s41598-022-07851-8, published online 09 March 2022

The original version of this Article contained an error in the Acknowledgments section.

“This study was supported by the Basic Science Research Program through the NRF funded by the Ministry of Science and ICT (NRF-2020R1A2C2005580), and the Ministry of Education (2019R1A2C1004575, WSC).”

now reads:

“This study was supported by the Basic Science Research Program through the NRF funded by the Ministry of Science and ICT (NRF-2020R1A2C2005580); MJS and (2019R1A2C1004575; WSC).”

The original Article has been corrected.

