# Supplementary material for: Neuroprotective effects of ATPase inhibitory factor 1 preventing mitochondrial dysfunction in Parkinson's disease
Source: Sci Rep. 2022 Mar 9;12:3874. doi: 10.1038/s41598-022-07851-8 (PMC8907304; doi:10.1038/s41598-022-07851-8)
Supplement: Supplementary file 1 — Supplementary Figures. [file 41598_2022_7851_MOESM1_ESM.docx]

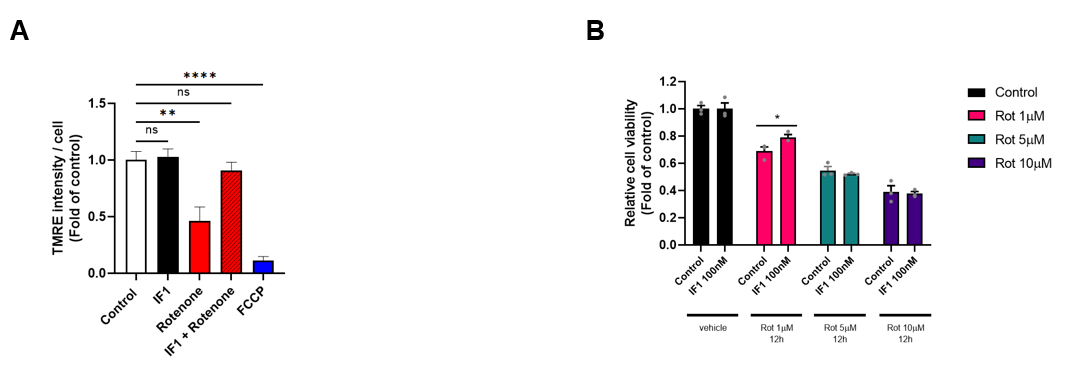


**Supplement S1. Early response to the IF1 administration in rotenone-treated SH-SY5Y cells**

**A,** SH-SY5Y cells were treated with 1 μM rotenone alone or combination of a specific concentration of IF1 (100 nM). After 6h incubation, mean fluorescence intensity of TMRE was normalized to the number of cells. **B**, SH-SY5Y cells were treated with rotenone (1, 5, 10 μM) with or without a specific concentration of IF1 (100 nM). After 12h incubation, cell viability was evaluated by MTT assay. Data are expressed as mean±SEM, n=3. * p<0.05, ** p<0.01, **** p<0.0001. ns; not significant. one-way ANOVA with a Tukey’s post-hoc analysis.


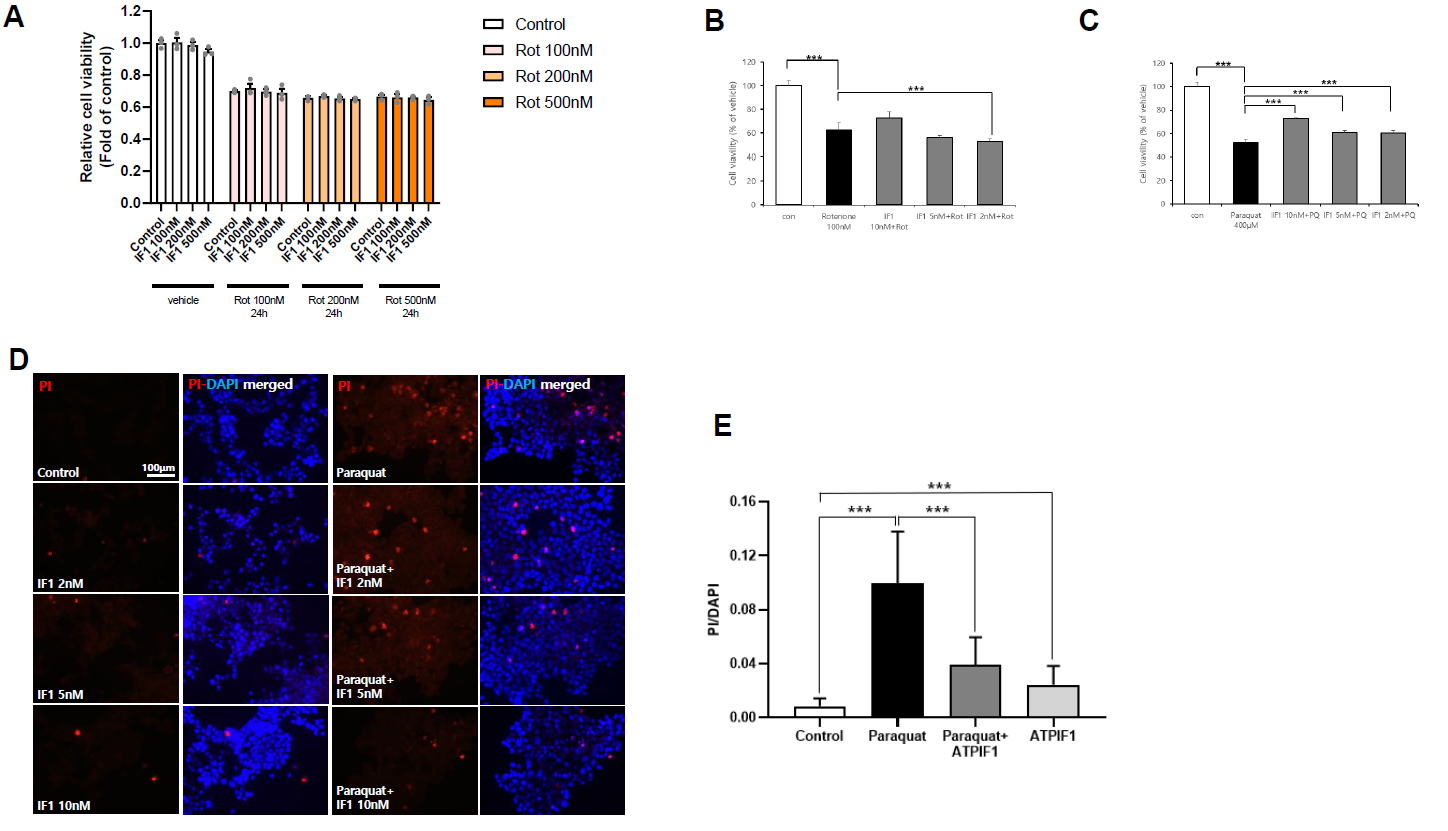


**Supplement S2. Effects of IF1 in pesticides-induced cell death in dopaminergic neural cell lines**

**A**, SH-SY5Y cells were treated with rotenone (100, 200, and 500 nM) with or without a specific concentration of IF1 (100, 200, and 500 nM). After 24h incubation, cell viability was evaluated by MTT assay. **B-E**, MN9D cells were treated with 100 nM rotenone **(B)** or 400 µM paraquat **(C-E)** alone or combination of a specific concentration of IF1 (2, 5, 10 nM). After 24h incubation, cell viability was analyzed by MTT assay **(B and C)** or PI assay **(D and E)**. **D**, Representative images of PI staining. Red: PI; Blue: Hoechst, Scale bar= 100 µm. **E**, A bar graph indicates the number of PI positive cells normalized to DAPI in each group. Data are expressed as mean±SEM, n=3. *** p<0.001. one-way ANOVA with a Tukey’s post-hoc analysis.


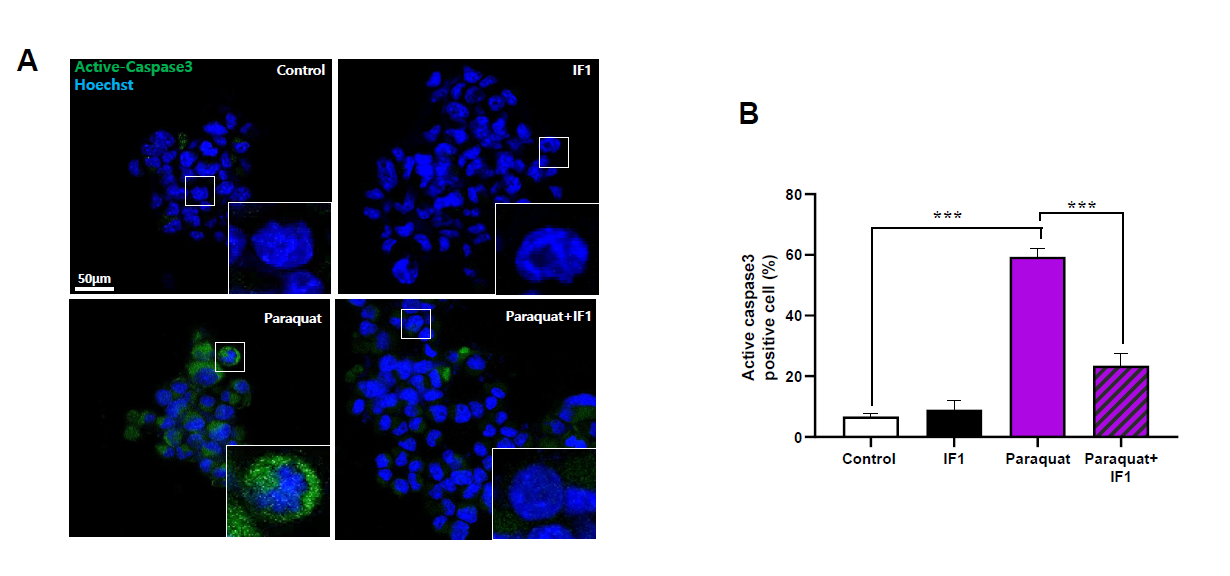


**Supplement S3. IF1 partially prevents paraquat-induced caspase-3 activation in MN9D cells**

MN9D cells were treated with 400 µM paraquat, 10 nM IF1 or a combination of both for 24h. **A**, Representative images of active-caspase 3 staining results. Red: active-caspase 3; Blue: Hoechst, Scale bar= 50 µm. **B**, A bar graph indicates the number of active-caspase-3 positive cells normalized to Hoechst in each group. Data are expressed as mean±SEM, n=3. *** p<0.001. one-way ANOVA with a Tukey’s post-hoc analysis.

**Uncropped Western Blots**

**Fig. 3E :**


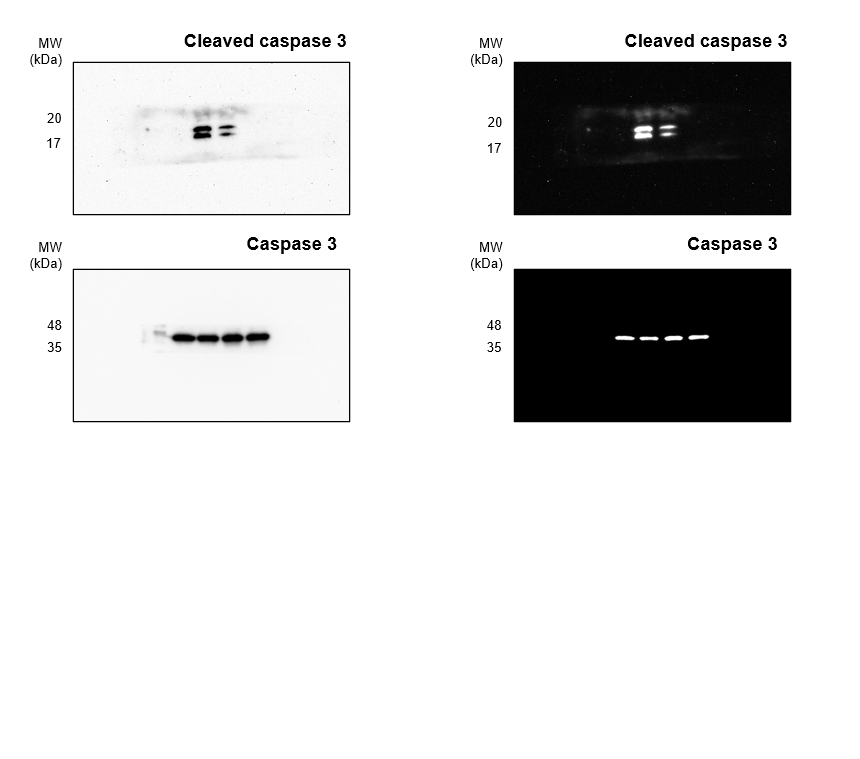

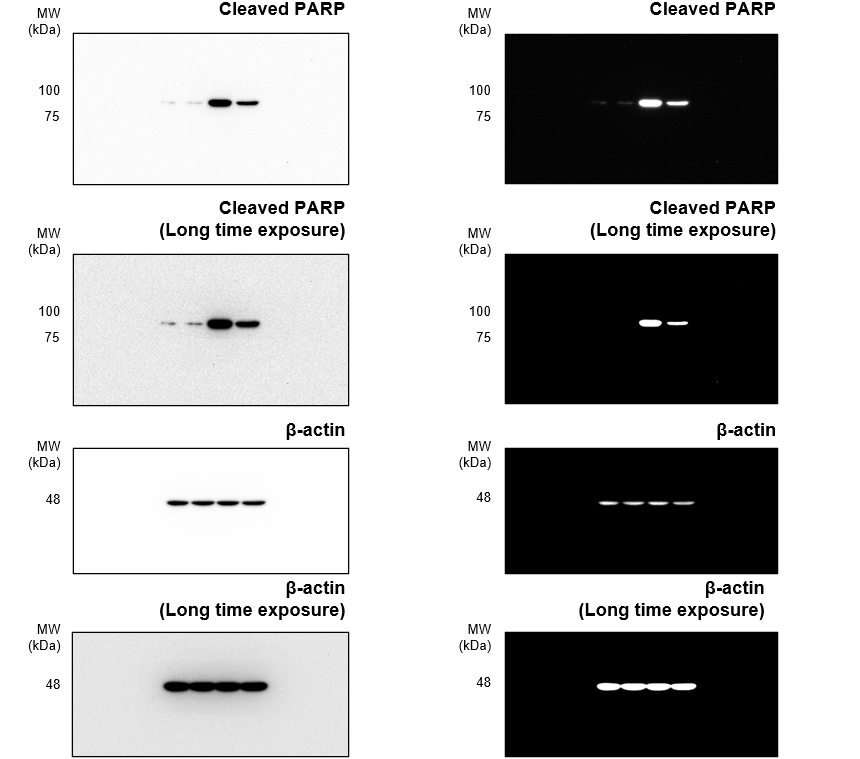


**(Long time exposure)**

**(Long time exposure)**

**(Long time exposure)**

**(Long time exposure)**

**Fig. 3F :**


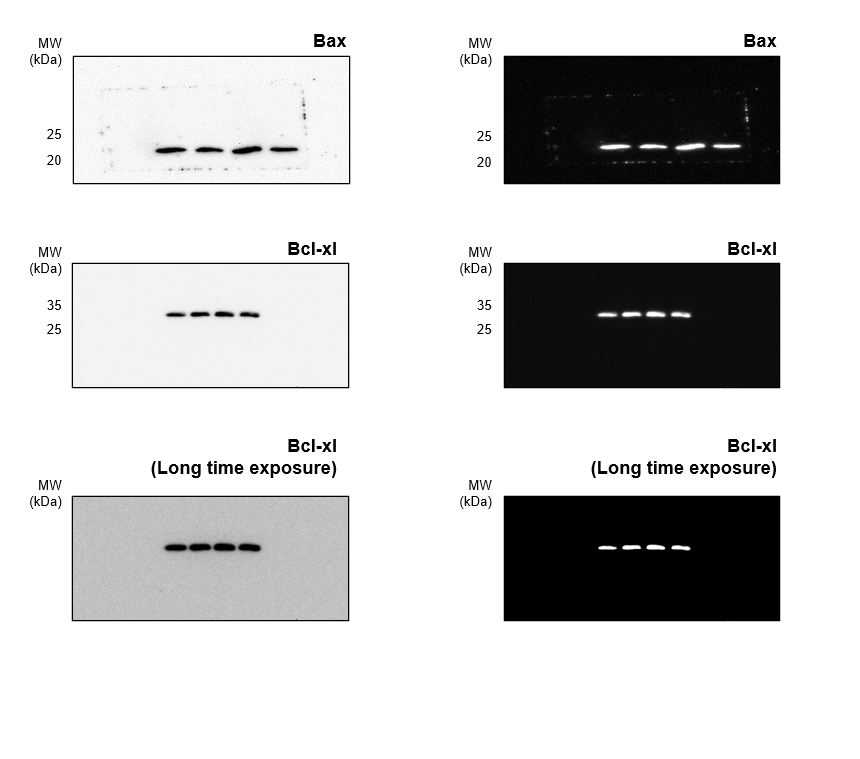

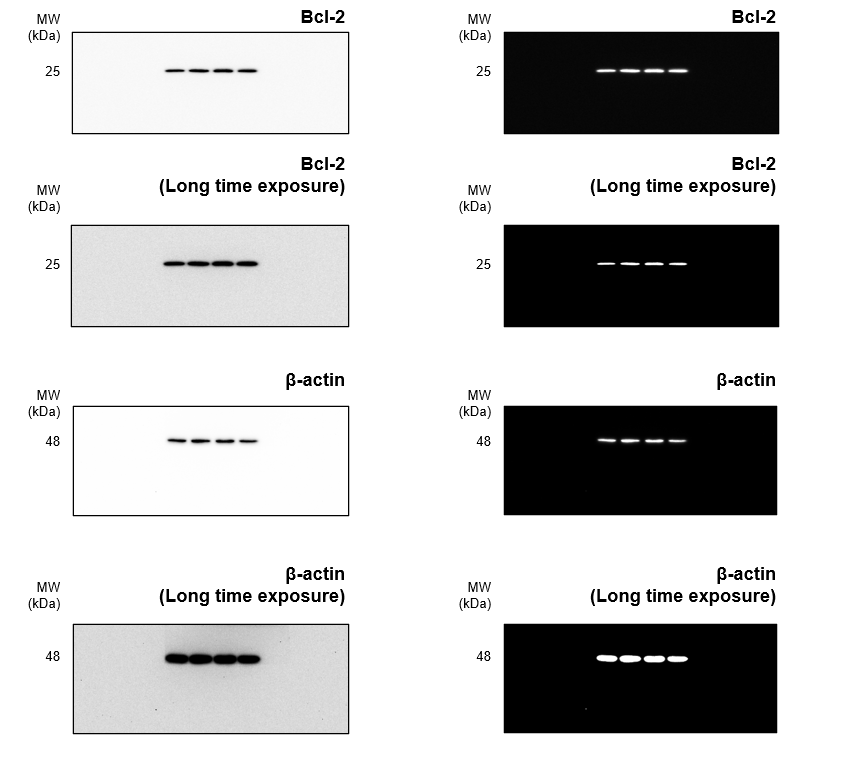


**(Long time exposure)**

**(Long time exposure)**

**(Long time exposure)**

**(Long time exposure)**

**(Long time exposure)**

**(Long time exposure)**
